# Age is not associated with intracranial haemorrhage in patients with mild traumatic brain injury and oral anticoagulation

**DOI:** 10.1186/s12952-016-0055-y

**Published:** 2016-06-01

**Authors:** Thomas C. Sauter, Stephan Ziegenhorn, Sufian S. Ahmad, Wolf E. Hautz, Meret E. Ricklin, Alexander Benedikt Leichtle, Georg-Martin Fiedler, Dominik G. Haider, Aristomenis K. Exadaktylos

**Affiliations:** Department of Emergency Medicine, Inselspital, University Hospital Bern, Freiburgstrasse, 3010 Bern, Switzerland; Centre of Laboratory Medicine, Inselspital, University Hospital Bern, Freiburgstrasse, 3010 Bern, Switzerland

**Keywords:** Mild traumatic brain injury, Anticoagulation, Age, Risk factor

## Abstract

**Background:**

Patients admitted to emergency departments with traumatic brain injury (TBI) are commonly being treated with oral anticoagulants. In contrast to patients without anticoagulant medication, no guidelines, scores or recommendations exist for the management of mild traumatic brain injury in these patients. We therefore tested whether age as one of the high risk factors of the Canadian head CT rule is applicable to a patient population on oral anticoagulants.

**Methods:**

This cross-sectional analysis included all patients with mild TBI and concomitant oral anticoagulant therapy admitted to the Emergency Department, Inselspital Bern, Switzerland, from November 2009 to October 2014 (*n* = 200). Using a logistic regression model, two groups of patients with mild TBI on oral anticoagulant therapy were compared — those with and those without intracranial haemorrhage.

**Results:**

There was no significant difference in age between the patient groups with (*n* = 86) and without (*n* = 114) intracranial haemorrhage (*p* = 0.078).

In univariate logistic regression, GCS (OR = 0.419 (0.258; 0.680)) and thromboembolic event as reason for anticoagulant therapy (OR = 0.486 (0.257; 0.918)) were significantly associated with intracranial haemorrhage in patients with mild TBI and anticoagulation (all *p* < 0.05). However, there was no association with age (*p* = 0.078, OR = 1.024 (0.997; 1.051)), the type of accident or additional medication with acetylsalicylic acid or clopidogrel ((both *p* > 0.05; 0.552 (0.139; 2.202) and 0.256 (0.029; 2.237), respectively).

**Conclusion:**

Our study found no association between age and intracranial bleeding. Therefore, until further risk factors are identified, diagnostic imaging with CCT remains necessary for mild TBI patients on oral anticoagulation of all ages, especially those with therapeutic anticoagulation because of thromboembolic events.

## Background

Traumatic brain injuries (TBI) are very common in emergency department admissions, with more than one million admissions per year in the USA alone [1, 2]. The case definitions of traumatic brain injury and even more so the definition of mild traumatic brain injury in the literature is very heterogeneous. We have now followed the definition of TBI as “an alteration in brain function, or other evidence of brain pathology, caused by an external force” [3].

Many studies have found that approx. 10–15 % of patients with head injuries and a Glasgow Coma Scale (GCS) of 15 exhibit bleeding that is detectable in the cranial computer tomogram (CCT) [4, 5]. In this population, fewer than 1 % of patients with bleeding are submitted to neurosurgical intervention for mild traumatic brain injury [4–7]. Although few cases of major intracranial haemorrhage are diagnosed via CCT imaging in this group of patients, this radiographic imaging is a standard diagnostic procedure. One American study has reported a marked increase in CCT in recent years [8]. Many guidelines and scores for the management of patients with mild traumatic brain injury try to limit the use of CCT to evidence based indications [4, 9].

For patients with risk factors such as anticoagulant medication, knowledge is limited, but approximately 20 % of these patients develop intracranial haemorrhage (ICH) [10]. Several studies have previously shown that in anticoagulated patients the risk for intracranial haemorrhage generally increases with age [11–13].

Neither the “Canadian CT Head Rule” nor the “New Orleans Criteria” apply to patients on anticoagulants [4, 9]. The clinical policy statement on “Neuroimaging and decision-making in adult mild traumatic brain injury in the acute setting” from the American College of Emergency Physicians (2008) states that the management of patients on anticoagulants is unclear and gives no specific recommendations [10]. In the 2014 review of the NICE guideline on head injury (No. 176), a CT head scan within 8 h of the injury is recommended for all patients on warfarin treatment, even without other clear indications for a CCT [14]. No data is provided for the observation period or whether these patients should be admitted to hospital. For patients on antiplatelet therapy, such as acetylsalicylic acid or clopidogrel, there are retrospective studies which suggest that there is an increased risk for intracranial bleeding in trauma patients [15, 16].

Another study investigated the impact of age in comparison to anticoagulation and found that more older patients needed neurosurgical intervention than patients taking anticoagulants [17].

In contrast to most studies on patients on oral anticoagulants (acetylsalicylic acid or clopidogrel) or on warfarin, more recent studies try to differentiate between oral anticoagulants and give specific recommendations. A recent study found that low dose acetylsalicylic acid is not associated with progression of initial haemorrhagic stroke or clinical deterioration [18]. However, another study detected an increased rate of neurosurgical interventions and clinical deterioration in patients on pre-trauma clopidogrel [19].

We hypothesized that age may not be associated with ICH in anticoagulated patients with mild TBI and therefore tested whether age as one of the high risk factors of the Canadian head CT rule is a risk factor for ICH in a patient population with mild traumatic brain injury on oral anticoagulants.

## Methods

This cross-sectional study included 260 patients on oral anticoagulant (OAC) therapy and with traumatic brain injury (TBI), defined as trauma to the head combined with loss of consciousness, amnesia and vegetative symptoms, who were admitted to the Emergency Department of the Inselspital between November 1, 2009, and October 31, 2014. Within this period, medical records were screened for “rivaroxaban” (Xarelto), “apixaban” (Eliquis) and “dabigatran” (Pradaxa) as well as “phenprocoumon” (Marcoumar). Patients with prehospital use of these medications and the documented diagnosis of traumatic brain injury were included. In order to minimise selection bias, we excluded patients transferred to our Level 1 hospital from other hospitals. Patients with an INR <2 under phenprocoumon at admission were also excluded.

Oral anticoagulation (OAC) was defined as medication with phenprocoumon or any new direct oral anticoagulants (DOAC), such as rivaroxaban, apixaban or dabigatran.

Data were collected on age, gender, nationality, primary outcome, traumatic brain injury, type of accident, medication, indication for oral anticoagulation, intracranial haemorrhage and location of bleeding, as well as the initial and lowest values of the Glasgow Coma Scale (GCS). Blood parameters monitored on admission were also registered (creatinine, INR).

The study protocol was approved and registered with the Ethics Committee of the Canton Bern, Switzerland. Because of the retrospective design of this cross-sectional study with anonymised data, a waiver for informed consent was issued.

### Statistical analysis

We compared patients on oral anticoagulant therapy with and without intracranial haemorrhage, with respect to age, GCS, creatinine and INR on admission by means of a Mann-Whitney *U* test. The risk for ICH in patients younger or older than age 65 was assessed via Chi-square test. Influences of different variables on intracranial haemorrhage were investigated with a logistic regression model for univariate tests.

All calculations were performed with the SPSS Statistics 21 (IBM Coorp.) program. A two tailed p value of less than 0.05 was considered statistically significant.

## Results

Overall 8886 patients had medical records with “Rivaroxaban”, “Apixaban”, “Dabigatran” or “Phenprocoumon”. Two hundred and sixty (260) of these patients had been admitted with the diagnosis of traumatic brain injury while currently on oral anticoagulation (OAC). The baseline characteristics of the patients included are shown in Table 1. As only a few of these patients were on the new DOACs (*n* = 19) rather than the classical phenprocoumon, statistical comparison of those groups was not meaningful.

**Table 1:** Baseline characteristics of all patients with traumatic brain injury taking OAC

Parameter	OAC	DOAC	Phenprocoumon
	*n* = 260	*n* = 19	*n* = 241
Age (years)	79.0 ± 11.3	73.7 ± 13.4	79.7 ± 11.1
Gender (male; female)	67 (25.8 %); 193 (74.2 %)	10 (52.6 %); 9 (47.4 %)	57 (23.7 %); 184 (76.3 %)
*Indication*			
Atrial fibrillation	130 (50.0 %)	8 (42.1 %)	122 (50.6 %)
Thromboembolic event	71 (27.3 %)	9 (47.4 %)	62 (25.7 %)
Prophylaxis post-surgery	2 (0.8 %)	2 (10.5 %)	0 (0 %)
Mechanical heart valve	16 (6.2 %)	0 (0 %)	16 (6.6 %)
Indication unclear	41 (15.8 %)	0 (0 %)	41 (17.0 %)
*Additional medication*			
Acetylsalicylic acid	16 (6.2 %)	1 (5.3 %)	15 (6.2 %)
Clopidogrel	8 (3.1 %)	2 (10.5 %)	6 (2.5 %)
*Intracranial haemorrhage general*	138 (53.1 %)	7 (36.8 %)	131 (54.4 %)
Epidural bleeding	9 (3.5 %)	0 (0 %)	9 (3.7 %)
Subdural bleeding	89 (34.2 %)	1 (5.3 %)	88 (36.5 %)
Subarachnoidal bleeding	39 (15.0 %)	3 (15.8 %)	36 (14.9 %)
Intracerebral bleeding	34 (13.1 %)	3 (15.8 %)	31 (12.9 %)

Definitions: Oral anticoagulation (OAC) = medication with phenprocoumon or direct oral anticoagulation (DOAC) with rivaroxaban, apixaban or dabigatran

Mean ± Standard deviation or absolute numbers (%)

**Fig. 1:**
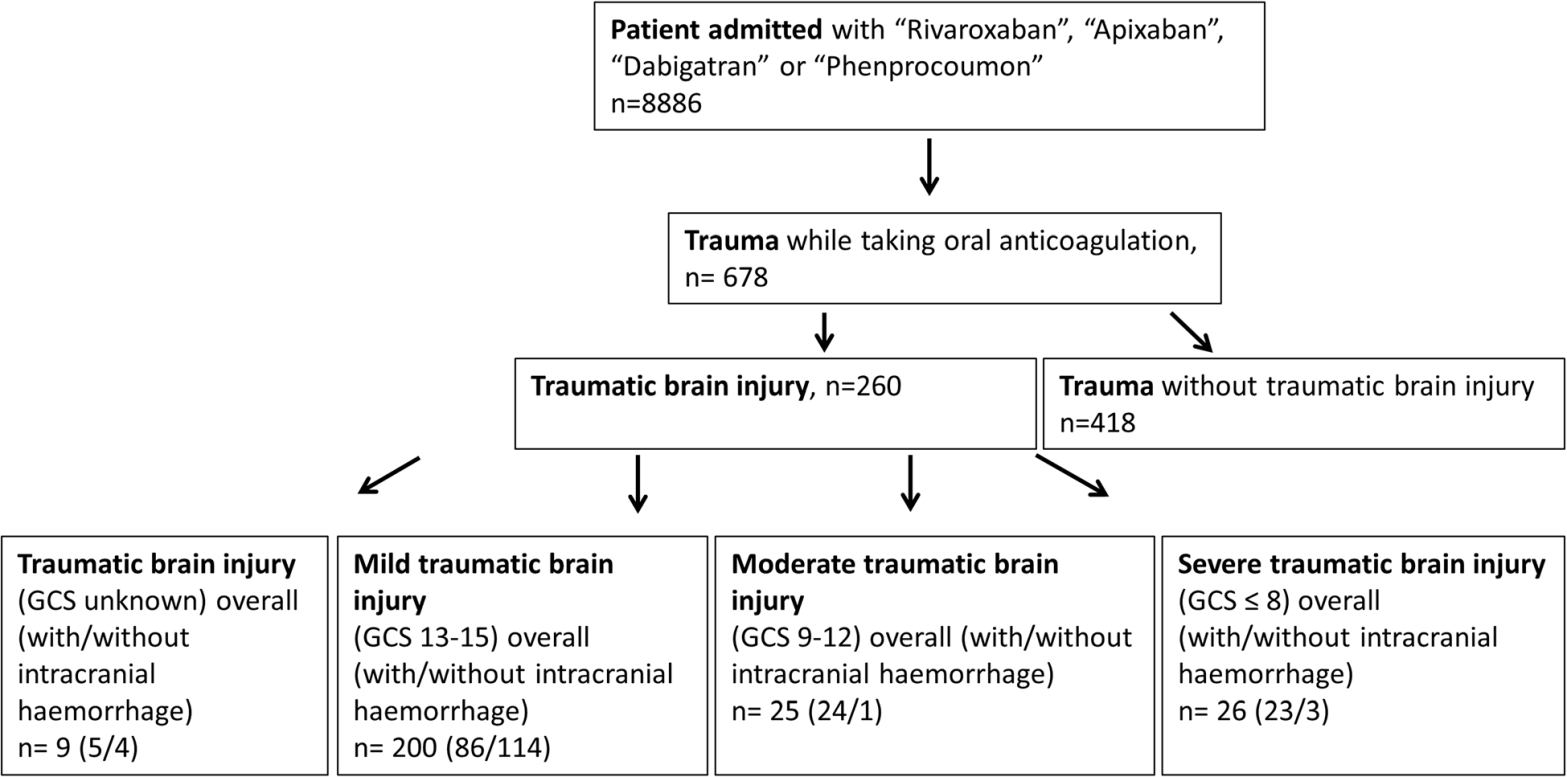
Patient assignment to the different subgroups of TBI

The numbers of our patients with traumatic brain injury and mild TBI (defined as GCS 13–15, *n* = 200), moderate TBI (GCS 9–12, *n* = 25) and severe TBI (GCS <9, *n* = 26) and also the number of patients with and without intracranial haemorrhage (ICH) are shown in Fig. 1. Overall, 8 (3.1 %) patients were on clopidogrel and 16 (6.2 %) patients on acetylsalicylic acid in combination with OAC therapy (Table 1). Table 1 lists the reasons for oral anticoagulation in patients with TBI.

For further evaluation we focused on patients with mild TBI, as patients with moderate and severe TBI need CT irrespective of age and coagulation status.

Mann-Whitney U tests were used to compare patient groups with (*n* = 86) and without (*n* = 114) ICH; the group with ICH exhibited lower GCS, as well as lower creatinine (Table 2, *p* < 0.05). There was no difference in age and INR between those groups (Table 2, *p* = 0.072, respectively *p* = 0.187). In particular there was no significant difference between mild TBI patients younger than 65 years and patients 65 years or older regarding ICH (*p* = 0.816). In the group of patients with mild TBI, 21 patients were younger than 65 years and 179 patients over 65 years.

**Table 2:** Comparison of patient groups with intracranial bleeding (*n* = 86) and without intracranial bleeding (*n* = 114) among patients with mild traumatic brain injury (*n* = 200 in total)

Parameter	Intracranial bleeding	No intracranial bleeding	p
	*n* = 86	*n* = 114	
Age	80.2 ± 10.3	77.3 ± 11.8	0.127
GCS	14.4 ± 0.7	14.8 ± 0.5	<0.001*
Creatinine	95.5 ± 41.5	103.7 ± 35.2	0.036*
INR	2.79 ± 0.97	2.66 ± 1.40	0.187

Mean; ±Standard deviation, Mann-Whitney U, *p* < 0.05*

*GCS* glasgow coma scale, *INR* international normalised ratio

Univariate logistic regression analysis revealed that GCS (*p* < 0.001, OR = 0.419 (0258; 0.680)) is associated with ICH in patients with mild TBI (Table 3). In addition, a thromboembolic event (*p* = 0.026, OR = 0.486 (0.257; 0.918)) as the reason for anticoagulant therapy was significantly associated with ICH (Table 3). In patients with mild TBI, ICH was not associated with age (*p* = 0.078, OR = 1.024 (0.997; 1.051)), the type of accident or additional medication with acetylsalicylic acid or clopidogrel (OR = 0.552 (0.139; 2.202) OR = 0.256 (0.029; 2.237), respectively) (Table 3).

**Table 3:** Associations of intracranial bleeding in patients with mild traumatic brain injury (*n* = 200) with different parameters in univariate analysis

Parameter	p	Odds ratio (confidence interval)
Age	0.078	1.024 (0.997; 1.051)
GCS	<0.001*	0.419 (0.258; 0.680)
Creatinine	0.214	0.994 (0.985; 1.003)
INR	0.528	1.092 (0.830; 1.438)
Gender	0.869	1.056 (0.554; 2.013)
*Indication*		
Atrial fibrillation	0.414	1.263 (0.721; 2.213)
Thromboembolic event	0.026*	0.486 (0.257; 0.918)
Mechanical heart valve	0.417	1.595 (0.516; 4.929)
*Additional medication*		
Acetylsalicylic acid	0.400	0.552 (0.139; 2.202)
Clopidogrel	0.218	0.256 (0.029; 2.237)
*Type of accident*		
Road traffic accident	0.934	0.960 (0.369; 2.501)
Fall	0.451	1.397 (0.586; 3.332)
Direct trauma	0.317	0.324 (0.036;2.948)

Univariate logistic regression, *p* < 0.05*

*GCS* glasgow coma scale, *INR* international normalized ratio

## Discussion

Our study demonstrates that age is not associated with ICH in patients on oral anticoagulants and with mild traumatic brain injury who were admitted as emergencies. However, thromboembolic events as indication for the oral anticoagulation are a risk factor for ICH in this patient group.

Age as a risk factor for ICH has been well studied in different populations with and without trauma and without anticoagulation: A recent meta-analysis of patients with non-traumatic and traumatic ICH in patients with non-ST-elevation myocardial infarction found that age is associated with an increased rate of ICH in this special patient population [20]. Large studies of TBI patients from Spain and Italy without anticoagulation therapy investigated different variables for the prediction of ICH and showed that age is associated with ICH [7, 21]. This is reflected in clinical guidelines and decision rules about the need for CCT scans in patients with minor TBI. The New Orleans criteria define age >60 years and the Canadian CT head rule age >65 years as strong risk factors and therefore recommend a CCT [4, 9]. In contrast to this, age was not associated with ICH in our study of mild TBI patients with OAC. Because of this, it is not possible to conclude that younger patients on anticoagulants are at low risk of ICH.

A further risk factor that could be demonstrated in our population was the indication of a thromboembolic event. It may be speculated that patients with this indication exhibit better compliance and are more closely monitored by their physicians than when the anticoagulation is only prescribed for prophylactic indications like atrial fibrillation. Patients with a prior thromboembolic event may take higher doses of anticoagulant and be at greater risk of ICH.

As this study was retrospective and involved the screening of medical records, it is possible that some cases were missed. Although we excluded secondary transport to our Level 1 university centre, selection bias from selective admission to our emergency department might be possible.

## Conclusion

Few factors are known to predict intracranial haemorrhage in patients with oral anticoagulation. Our study showed no association between age and intracranial bleeding. Therefore, diagnostic imaging with CCT is still necessary for patients of all ages with mild TBI, especially with therapeutic anticoagulation because of thromboembolic events as nearly half of anticoagulated patients with mild TBI had ICH on CCT.

## Abbreviations

CCT: cranial computer tomogram
DOAC: direct new oral anticoagulants
GCS: Glasgow coma scale
ICH: intracranial haemorrhage
INR: international normalised ratio
OAC: oral anticoagulant
TBI: traumatic brain injury
